# The α_1_- and β_1_-Subunits of Nitric Oxide-Sensitive Guanylyl Cyclase in Pericytes of Healthy Human Dental Pulp

**DOI:** 10.3390/ijms26010030

**Published:** 2024-12-24

**Authors:** Yüksel Korkmaz, Galyna Pryymachuk, Mechthild M. Schroeter, Behrus Puladi, Nadin Piekarek, Sarah Appel, Wilhelm Bloch, Jan-Wilm Lackmann, James Deschner, Andreas Friebe

**Affiliations:** 1 Department of Periodontology and Operative Dentistry, University Medical Center of the Johannes Gutenberg University Mainz, 55131 Mainz, Germany; james.deschner@uni-mainz.de; 2 Institute of Anatomy, Brandenburg Medical School Theodor Fontane, 14770 Brandenburg an der Havel, Germany; galyna.pryymachuk@mhb-fontane.de; 3 Department of Anatomy I, University of Cologne, 50937 Cologne, Germany; 4 Center for Physiology and Pathophysiology Faculty of Medicine and University Hospital Cologne, 51109 Cologne, Germany; mechthild.schroeter@uni-koeln.de; 5 Department of Oral and Maxillofacial Surgery, University Hospital RWTH Aachen, RWTH Aachen University, 52074 Aachen, Germany; bpuladi@ukaachen.de; 6 Experimental Medicine, Faculty of Medicine and University Hospital Cologne, University of Cologne, 51109 Cologne, Germany; nadin.piekarek@uk-koeln.de; 7 Department of Pediatrics and Adolescent Medicine, Faculty of Medicine and University Hospital Cologne, University of Cologne, 50937 Cologne, Germany; sarah.appel@uk-koeln.de; 8 Department of Molecular and Cellular Sport Medicine, German Sport University Cologne, 50933 Cologne, Germany; w.bloch@dshs-koeln.de; 9 Cluster of Excellence Cellular Stress Responses in Aging-Associated Diseases, University of Cologne, 50931 Cologne, Germany; jan-wilm.lackmann@uni-koeln.de; 10 Institute of Physiology, University of Würzburg, 97070 Würzburg, Germany; andreas.friebe@uni-wuerzburg.de

**Keywords:** pericytes, nitric oxide (NO), NO-GC, NO-GC α_1_-subunit, NO-GC β_1_-subunit, dental pulp, reparative tertiary dentin matrix, dental pulp fibrosis

## Abstract

Nitric oxide-sensitive guanylyl cyclase (NO-GC) is a heterodimeric enzyme with an α- and a β-subunit. In its active form as an α_1_β_1_-heterodimer, NO-GC produces cyclic guanosine-3′,5′-monophophate (cGMP) to regulate vasodilation and proliferation of vascular smooth muscle cells (VSMCs). In contrast to VSMCs, only a few studies reported on the expression of the NO-GC α_1_β_1_-heterodimer in human pericytes. Since NO-GC is a marker for platelet-derived growth factor-β (PDGFRβ)-positive pericytes, we investigated whether NO-GC is expressed in its active α_1_β_1_-heterodimer in pericytes of healthy human dental pulp. In our previous studies, we developed and validated an antibody against the α_1_-subunit of human NO-GC. Here, we developed a new antibody against the β_1_-subunit of human NO-GC and validated it by immunoblot, mass spectrometry, and immunohistochemistry on tissue samples from humans and NO-GC knockout (GCKO) mice. Using both antibodies, we detected α_1_- and β_1_-subunits of NO-GC in pericytes of pre-capillary arterioles, capillaries, and post-capillary venules in dental pulp of decalcified and non-decalcified human molars. We concluded that NO-GC as an active α_1_β_1_-heterodimer may be involved in the regulation of vascular permeability, vascular stability, organ homeostasis, and organ regeneration in healthy human dental pulp.

## 1. Introduction

The inter- and intracellular signaling molecule nitric oxide (NO)-sensitive guanylyl cyclase (NO-GC) is a heterodimeric enzyme with α and β subunits [1]. On the genomic level, two different α-subunits (α_1_ and α_2_) and two different β-subunits (β_1_ and β_2_) of NO-GC exist, but only the α_1_β_1_- and α_2_β_1_-heterodimers have been shown to form catalytically active enzymes [1]. The binding of NO to the heme group of NO-GC activates the enzyme, leading to increased production of cyclic guanosine-3′,5′-monophophate (cGMP) from guanosine-5′-triphosphate (GTP) [1]. NO-GC is known to regulate a variety of functions, including vasodilation [2], inhibition of vascular smooth muscle cell (VSMC) migration and proliferation [3], leukocyte recruitment, platelet aggregation [4], and the modulation of neurotransmission [5].

The vasculature consists of a branching system of blood vessels including arteries, pre-capillary arterioles, capillaries, post-capillary venules, and veins that promote blood circulation between the heart, lungs, and target tissues [6,7]. With a diameter of 5–10 μm, the capillaries are the smallest blood vessels, lacking a continuous layer of VSMCs [8]. VSMCs encircle the endothelial cells of arteries, arterioles, venules, and veins, whereas pericytes are embedded in the basement membrane of capillaries, pre-capillary arterioles, and post-capillary venules [6,9]. At their sites, VSMCs and pericytes regulate vasodilation and vasoconstriction controlling the permeability and stability of blood vessels [8,10].

The odontoblast-like cells form the reparative tertiary dentin to protect the exposed pulp against carious bacteria [11,12]. It has been shown that pericytes are progenitor cells for odontoblast-like cells in dental pulp [13]. The deep carious lesions that destroy terminally differentiated odontoblasts trigger stimuli to which pericytes respond [14]. As a consequence, resident pericytes migrate to the site of the lesion and differentiate into odontoblast-like cells [14,15]. It has also been shown that, in different organs, pericytes differentiate into myofibroblasts and form a fibrotic extracellular matrix in response to inflammation [16]. Although the main cellular and molecular mechanisms of fibrosis in other organs are adequately understood [16], knowledge about the mechanisms of fibrosis in dental pulp is sparse. Only age-related fibrotic changes in dental pulp have been described [17,18].

Regarding the inhibition of VSMC proliferation and relaxation of blood vessels, the role of NO-GC has been extensively studied [2,3]. However, there are only a few studies on the expression of NO-GC in pericytes, e.g., in retina [19], skeletal muscle [20], liver [21,22], kidney [21], and lung [23,24]. In capillaries, activation of NO-GC in pericytes stabilizes the interactions between endothelial cells and pericytes [24]. NO-GC has an inhibitory effect on endothelial cell permeability and pericyte migration, preventing inflammation and fibrosis [24,25]. This could indicate critical functions for NO-GC in the migration and differentiation of pericytes to odontoblast-like cells and to myofibroblasts in inflamed human dental pulp.

Although several markers for pericytes have been described, the differential expression of these proteins in pericytes limits the use of a single protein as a pan-pericyte marker [23,26]. Using lineage tracing studies in combination with double staining, it has been shown that NO-GC is a marker for platelet-derived growth factor-β (PDGFRβ)-positive pericytes [23]. Under physiological conditions, NO-GC is active as α_1_β_1_-heterodimer. Therefore, we investigated the α_1_- and β_1_-subunits of NO-GC in pericytes of healthy human dental pulp. To this purpose, we raised an antibody against the α_1_-subunit of human NO-GC in rabbits [27]. In the present study, we developed a new antibody against the β_1_-subunit of human NO-GC and validated it by immunoblot, mass spectrometry, and immunohistochemistry on tissue samples from humans and NO-GC-knockout mice (GCKO).

## 2. Results

### 2.1. Characterization of the Healthy Human Dentin–Pulp Complex

In HE-stained sections of healthy dentin–pulp complex, primary dentin, secondary dentin, predentin, the odontoblast layer, the cell-free and cell-rich layers, and the subodontoblastic plexus with nerve fibers and blood vessels were identified in a cellular and structural order (Figure S1A,B in Supplementary Materials). Numerous pulp cells were observed in healthy dental pulp (Figure S1A,B in Supplementary Materials). In addition, several dental pulp cells were found around the blood vessels and nerve fibers of healthy dental pulp (Figure S1B in Supplementary Materials).

It has been shown that there are no mast cells in healthy dental pulp, whereas there are numerous human leukocyte antigen-DR isotype (HLA-DR) and some cluster of differentiation 68 (CD68)-positive cells [28]. Thus, the consecutive sections of HE staining were also examined for mast cell tryptase (MCT) and HLA-DR expression. No MCT-positive cells were detected in healthy dental pulp (Figure S1C,D in Supplementary Materials), whereas HLA-DR was found in numerous cells of the dentin–pulp complex (Figure S1E,F in Supplementary Materials). Following histopathological characterization of healthy dentin–pulp complex sections, the α_1_- and β_1_-subunits of NO-GC were examined in consecutive MCT-negative sections, which also showed no degeneration of odontoblasts.

### 2.2. Specificity of NO-GC α_1_ and β_1_ Antibodies

#### 2.2.1. Specificity of NO-GCα_1_ Antibody

The specificity of the polyclonal NO-GCα_1_ antibody developed in rabbits against the human α_1_-subunit of NO-GC (EP101278: ID0490; Eurogentec, Seraing, Belgium) was characterized by immunohistochemistry and immunoblotting as described previously [27].

#### 2.2.2. Specificity of NO-GCβ_1_ Antibody

**Figure 1:**
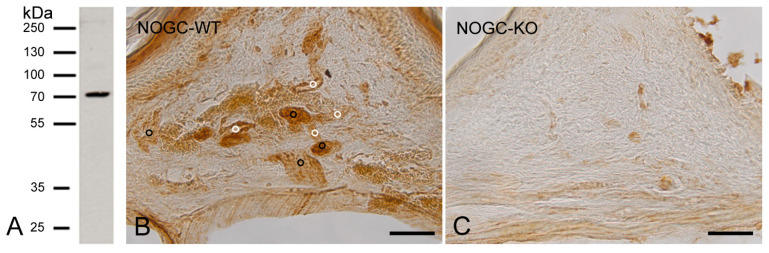
The specificity of the rabbit anti human NO-GCβ_1_ antibody (EP111806: ED11005; Eurogentec, Seraing, Belgium). In protein extracts of human placenta, the NO-GCβ_1_ antibody detected a single protein band with an apparent molecular mass of about 70 kDa corresponding to that of the β_1_-subunit of human NO-GC (**A**). A strong immunohistochemical signal for the NO-GCβ_1_ is detected in blood vessels (white points) and nerve fibers (black points) of the oral mucosa from WT-mice (**B**). Only weak unspecific staining was detected in GC-KO tissue (**C**). Scale bars: B,C = 50 μm.

As the NO-GCβ_1_ antibody was developed against a human peptide sequence, we tested the specificity of the antibody in Western blots on human placenta tissue samples. In human placenta protein extracts, the antibody detected a prominent band with an apparent molecular mass of about 70 kDa (Figure 1A). The corresponding protein band was analysed by mass spectrometry (Figure S2 in Supplementary Materials) and the resulting peptides were found to be specific for the β_1_-subunit of human NO-GC. Mass spectrometry analysis of the excised band revealed five peptides of the β_1_-subunit of NO-GC, corresponding to a coverage of 9.4% of the β_1_-subunit of the NO-GC protein (Figure S2 in Supplementary Materials).

In blood vessels and in nerve fibers of the oral mucosa from WT-mice, we detected a high expression of NO-GCβ_1_ (Figure 1B). In contrast, only weak unspecific staining was detected in the oral mucosa of GCKO mice (Figure 1C).

### 2.3. Expression of NO-GCα_1_ and β_1_ Subunits in Pericytes of Decalcified Dental Pulp

We investigated the effects of decalcification on the expression of the NO-GCα_1_ and NO-GCβ_1_ in pericytes of human dental pulp. For this purpose, we decalcified six molars from six different patients.

**Figure 2:**
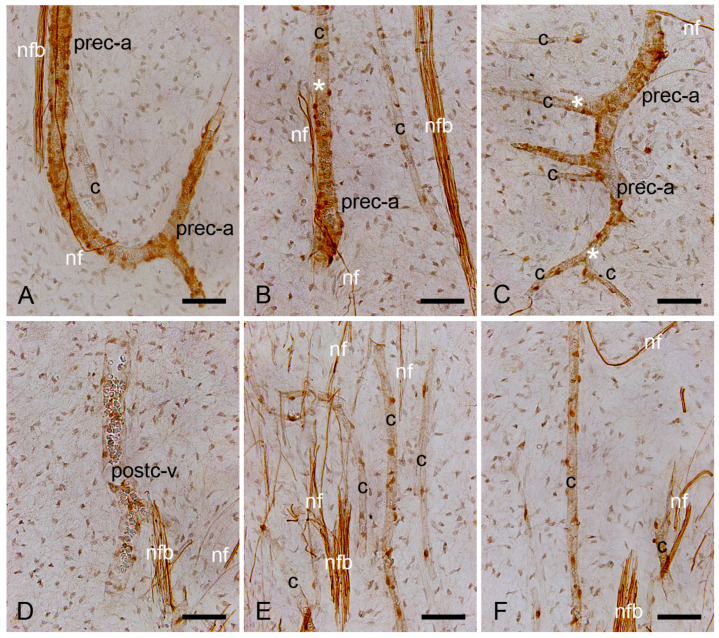
Expression of NO-GCβ_1_ in pericytes of decalcified healthy human dental pulp. In the dental pulp, a NO-GCβ_1_ signal was detected in pericytes, and a moderate signal was detected within the wall (vascular smooth muscle cells) of pre-capillary arterioles (prec-a) (**A**–**C**). In the capillary branches (c) of the same vessels, only the pericytes were positive for NO-GCβ_1_ (**B**,**C**). The asterisks indicate the transition areas from pre-capillary arterioles (prec-a) to capillaries (c) (**B**,**C**). Expression of NO-GCβ_1_ in pericytes of post-capillary venules (postc-v) (**D**) and capillaries (c) (**E**,**F**). Note that NO-GCβ_1_ was also expressed in nerve fiber bundles (nfb) and in fine nerve fibers (nf) of human dental pulp (**A**–**F**). Scale bars: A–F = 50 μm.

In the decalcified healthy human dental pulp sections, using the avidin–biotin–peroxidase complex method, NO-GCβ_1_ was detected in pericytes surrounding pre-capillary arterioles (Figure 2A–C), capillaries (Figure 2B–F), and post-capillary venules (Figure 2D).

In addition, nerve fiber bundles and fine nerve fibers in human dental pulp were also positive for NO-GCβ_1_ (Figure 2A–F).

To test the expression of NO-GCβ_1_ in nerve fibers of the dentin–pulp complex, we performed co-localization incubations with the NO-GCβ_1_ antibody and the neuronal marker NF200, which recognizes myelinated Aβ and Aδ nerve fibers. We confirmed the results of our avidin–biotin–peroxidase complex staining and found a co-localization of NO-GCβ_1_ with NF200 in nerve fibers of decalcified human dental pulp (Figure S3A–D in Supplementary Materials).

**Figure 3:**
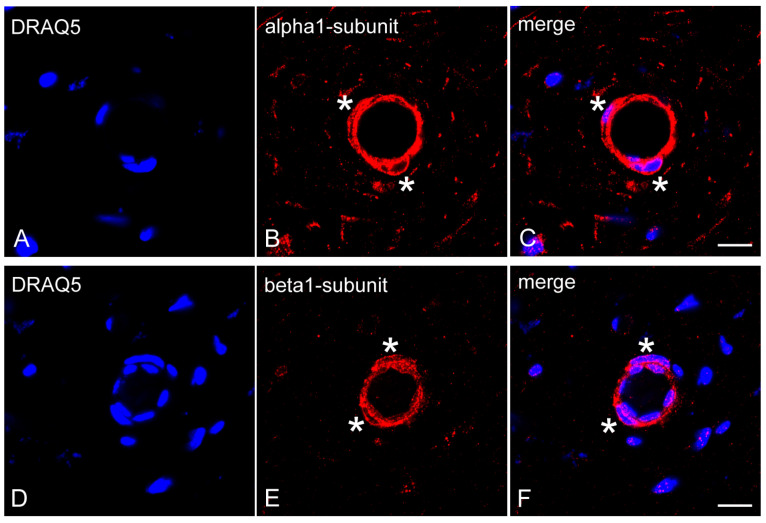
Expression of NO-GCα_1_ and NO-GCβ_1_ in pericytes of decalcified healthy dental pulp. Cell nuclei were visualized with DRAQ5 staining (**A**,**C**,**D**,**F**). Transverse sections of dental pulp show NO-GCα_1_ (**B**,**C**) and NO-GCβ_1_ (**E**,**F**) expressions in VSMCs (the wall of the pre-capillary arterioles) and in pericytes (embedded in the wall of the pre-capillary arterioles; asterisks). Scale bars: A–F = 10 μm.

Using immunofluorescence methods on dental pulp sections of decalcified human molars, NO-GCα_1_ (Figure 3A–C) and NO-GCβ_1_ (Figure 3D–F) were detected in pericytes of the capillaries, pre-capillary arterioles, and post-capillary venules.

### 2.4. Expression of NO-GCα_1_ and β_1_ Subunits in Pericytes of Non-Decalcified Dental Pulp

We tested the expression of NO-GCα_1_ and NO-GCβ_1_ in the pulp pericytes of non-decalcified human molars in order to compare the results with those of decalcified molars. For this purpose, we obtained the pulp tissue of three different molars from three different patients.

**Figure 4:**
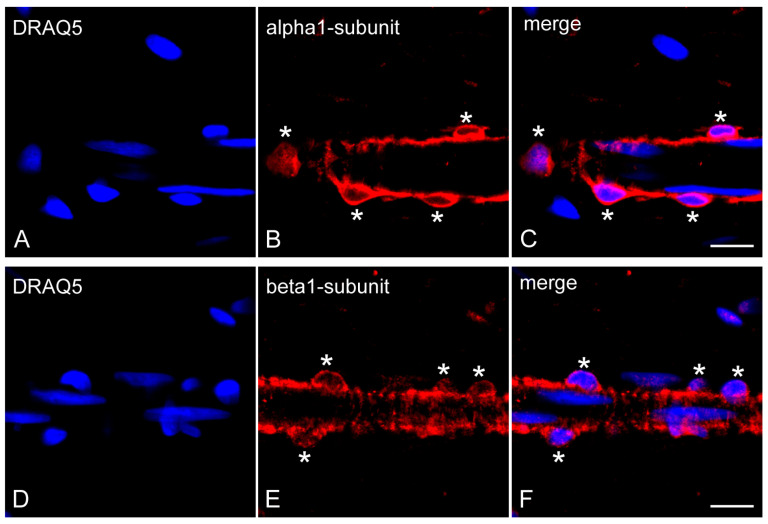
Expression of NO-GCα_1_ and NO-GCβ_1_ in pericytes of non-decalcified healthy dental pulp. Cell nuclei were visualized with DRAQ5 staining (**A**,**C**,**D**,**F**). Longitudinal sections of dental pulp show NO-GCα_1_ (**B**,**C**) and NO-GCβ_1_ (**E**,**F**) expressions in VSMCs (the wall of the pre-capillary arterioles) and in pericytes (embedded in the wall of the pre-capillary arterioles; asterisks). Scale bars: A–F = 10 μm.

In non-decalcified dental pulp of human molars, NO-GCα_1_ was found in the pericytes of the capillaries, the pre-capillary arterioles, and the post-capillary venules (Figure 4A–C). NO-GCβ_1_ was also detected in the pericytes of capillaries, pre-capillary arterioles, and post-capillary venules of non-decalcified human molar dental pulp (Figure 4D–F).

### 2.5. Immunohistochemical Controls

In control incubations of the avidin–biotin–peroxidase complex (Figure S4A–D in Supplementary Materials) and immunofluorescence methods, no immunohistochemical staining was found in cells of the human dentin–pulp complex.

### 2.6. Statistical Analysis of the Fluorescence Intensity of NO-GCα_1_ and NO-GCβ_1_ in Pericytes of Decalcified and Non-Decalcified Dental Pulp

**Figure 5:**
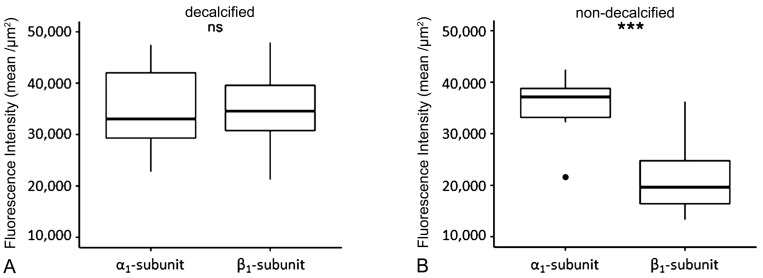
Analysis of immunohistochemical signal intensities of the α_1_- and β_1_-subunits of NO-GC in pericytes of the dental pulp from decalcified (*n* = 6) (**A**) and non-decalcified (*n* = 3) (**B**) molars. The black circle is an outlier. ns equals (*p* ≥ 0.05) and *** equals (*p* < 0.001).

In pericytes of decalcified dental pulp, comparison of the immunofluorescence-signal intensities between the NO-GCα_1_ and NO-GCβ_1_ antibodies showed no significant differences (Figure 5A). However, NO-GCα_1_ was significantly higher expressed in pericytes of non-decalcified dental pulp than NO-GCβ_1_ (Figure 5B).

## 3. Discussion

The dental pulp is supplied by end-arteries and end-veins through apical foramen, and it is located in a rigid calcified extracellular dentin matrix. Therefore, dental pulp has a limited ability to expand in response to inflammation induced by caries [29,30]. VSMCs and pericytes regulate by vasodilation and vasoconstriction the permeability and stability of blood vessels [8,10]. Here, we detected α_1_- and β_1_-subunits of NO-GC in pericytes of pre-capillary arterioles, capillaries, and post-capillary venules in healthy human dental pulp. Our results indicate an important function of the α_1_β_1_-heterodimer of NO-GC in pericytes regulating the vascular integrity and tissue homeostasis in human dental pulp under physiological and inflammatory conditions.

The pulp and dentin together form a dentin–pulp unit called the dentin–pulp complex. The odontoblast cell bodies are located in the peripheral pulp, while the odontoblast processes extend within the odontoblast tubules to near the enamel–dentin junction. To determine the depth of the carious lesion in the dentin to the pulp, and, thus, to make a histopathological diagnosis of the healthy and carious dentin–pulp unit, the teeth must be decalcified. The dentin–pulp complex must also be fully sectioned and histopathologically characterized at equal intervals. In our studies, we completely sectioned each human molar from the point-like opening of the dentin–pulp complex to the point-like end of the dentin–pulp complex and diagnosed it as healthy or pathological at 720 µm intervals (every 24th section; section thickness: 30 µm) on 5–6 levels. If the dentin–pulp complex is histopathologically healthy at all levels, the tooth is considered healthy. However, if there is a pathology in a level (e.g., odontoblast degeneration, denticle, hyaline degeneration, sterile inflammation, etc.), the tooth is not considered healthy.

In pericytes of decalcified dental pulp, signal intensities of the α_1_- and β_1_-subunits of NO-GC were similar without significant differences. In pericytes of non-decalcified dental pulp, the signal intensity of the α_1_-subunit of NO-GC was significantly higher than that of the β_1_-subunit. These results suggest that the α_1_ subunit of NO-GC is highly expressed in pericytes of non-decalcified dental pulp compared to the β_1_-subunit of NO-GC. Therefore, it is possible that the observed difference is probably due to the influence of decalcification on the antibody affinity for the β_1_-subunit of the NO-GC.

In capillaries, pericytes have cell–cell surface contacts with endothelial cells via gap and adherens junctions [9,26]. NO is produced in endothelial cells and diffuses into neighboring pericytes [31]. In addition, pericytes are known to produce endogenous NO [10,24]. In VSMCs, the receptor of NO is NO-GC, which induces the formation of cGMP to trigger the relaxation of VSMCs in α_1_β_1_-heterodimers [1,2]. The expression of both the α_1_- and β_1_-subunits of NO-GC in pericytes of healthy human dental pulp indicates that NO-GC is expressed in pericytes in its active form as α_1_β_1_-heterodimer. It is therefore plausible to assume that, in pericytes, paracrine (from endothelial cells) and autocrine (from pericytes) generated NO induces the activation of NO-GC in the α_1_β_1_-heterodimer, in order to produce cGMP. Thus, the NO-cGMP signaling cascade may regulate pericytes to maintain vascular stability, blood flow, and vascular permeability in healthy human dental pulp.

The redox status of iron of NO-GC, Fe^2+^ under physiological conditions and Fe^3+^ under inflammatory conditions, is crucial for the activity of NO-GC in a cell [2,32]. During inflammation, O_2_^−^ and ONOO^-^ (peroxynitrite) oxidize Fe^2+^ to Fe^3+^ within the β_1_-subunit of NO-GC, leading to the insensitivity of NO-GC to NO [2,32]. Since higher levels of O_2_^−^ and ONOO^-^ have been detected in inflamed dental pulp [33], we speculate that in the oxidized state, during inflammation of the dental pulp, NO-GC could be NO-insensitive. Thus, in pericytes of inflamed dental pulp, NO- and hem-independent pharmacological activation of NO-GC may be considered as a therapeutic strategy for manipulating pericyte cell functions.

Genetic lineage tracing studies, performed with cre-recombinase mouse lines, provide in vivo evidence that a subpopulation of odontoblast progenitors derive from dental pulp pericytes [13,34]. Pericytes are pulpal stem cells [13,35]. It is known that human dental pulp stem cells develop a pro-fibrotic phenotype under inflammatory conditions [36,37]. It has been shown that activation of NO-GC in pericytes stabilizes the interactions between endothelial cells and pericytes in capillaries. Under conditions of acute inflammation, this pericyte–endothelial cell interaction is disrupted, leading to an increase in vascular permeability [24]. In the bleomycin-induced lung fibrosis model, GCKO mice showed enhanced lung fibrosis and inflammation compared to WT mice, indicating that NO-GC has inhibitory effects on fibrosis and inflammation [25]. In the case of pulp inflammation, it is likely that pharmacological activation of the NO-cGMP signaling cascade by NO- and hem-independent activators of NO-GC (in pulp capping materials) could reduce vascular permeability and inhibit inflammation-induced pericyte migration and differentiation to the myofibroblasts and odontoblasts-like cells.

In conclusion, we found that pericytes of healthy dental pulp express the α_1_- and β_1_-subunits of NO-GC, which are required for activity of NO-GC in α_1_β_1_-heterodimer form. The NO- and hem-dependent activation of NO-GC in α_1_β_1_-heterodimer form in pericytes of healthy human dental pulp suggest an important role for NO-GC in the regulation of vascular permeability, vascular stability, organ homeostasis and organ regeneration.

Here, there are limitations to our study. We did not investigate the functional role or mechanisms of the NO-GC α_1_β_1_-heterodimer in the migration and differentiation of pericytes to odontoblast-like cells and to myofibroblasts in inflamed human dental pulp. Our further studies on the activation or inhibition of the NO-GC α_1_β_1_ heterodimer in pericytes of inflamed human dental pulp should clarify the role of NO-GC in pulp fibrosis and reparative tertiary dentin matrix formation.

## 4. Materials and Methods

### 4.1. Clinical Evaluation and Collection of Human Molars

The molars were healthy, non-carious, clinically asymptomatic, and showed no pain on stimuli-induced testing and percussion.

The Human Ethics Committee of the Heinrich-Heine University Düsseldorf approved the collection of human third molars extracted due to orthodontic treatment (Nr.: 2980).

### 4.2. Tissue Preparation

The clinically healthy diagnosed (*n* = 11) non-carious third molars were extracted from different patients (*n* = 11) who had undergone orthodontic extraction treatment. The molars were immersion-fixed in a fixative containing 4% paraformaldehyde and 0.2% picric acid in 0.1 M phosphate-buffered saline (PBS), pH 7.4, for 48 h. The molars were demineralized in 4 M formic acid for 21 days. The molars were washed in 0.1 M PBS for 72 h and cryoprotected with 30% sucrose solution in 0.1 M PBS, pH 7.4, for 48 h. The molars were then cryo-embedded, stored at −82 °C and cryo-sectioned on a cryostat at 30 μm.

The clinically healthy diagnosed (*n* = 5) non-carious third molars were extracted from different patients (*n* = 5), who had undergone orthodontic extraction treatment. The molars were mesio-distal drilled using water-cooled diamond drills up to the dentin half, so that the teeth could be divided into two parts by elevators. The pulp was immediately removed from the teeth by an excavator and immersion-fixed. Without decalcification, the samples were processed by the steps described above for the decalcified teeth.

### 4.3. Histopathological Evaluation of Healthy and Inflamed Human Dental Pulp

Molars and pulp tissues were completely sectioned for histologic examination. Pulpal inflammation is determined by histopathological diagnosis. Therefore, 5–7 sections (using the same intervals) per molar were stained with hematoxylin and eosin (HE staining). The results of HE staining were characterized by histopathological diagnoses. Afterwards, consecutive sections of HE staining were analyzed for expressions of MCT- and HLA-DR by immunohistochemistry. According to the histopathological diagnosis, 6 decalcified molars and 3 non-decalcified pulp tissues were found to be healthy. As in other molars where sterile inflammation of the pulp, vacuolar degeneration of the odontoblasts, degeneration of the pulp or denticle formation was observed, these molars were not considered to be healthy.

### 4.4. Specificity of NO-GC α_1_ and β_1_ Subunit Antibodies

#### 4.4.1. Characterization of NO-GCα_1_ Antibody

We have previously developed a specific polyclonal antibody against the human α_1_-subunit of NO-GC raised in rabbits (EP101278: ID0490; Eurogentec, Seraing, Belgium). The specificity of this antibody was verified by immunohistochemistry and immunoblotting [27].

#### 4.4.2. Development and Characterization of NO-GCβ_1_ Antibody

The antibody against the human β_1_-subunit of NO-GC was generated in a rabbit (SA7023) by immunization with a synthetic peptide (EP111806: ED11005) containing the C-terminal domain (CSRKNTGTEETKQDDD) of the human β_1_-subunit (Eurogentec). The rabbit was immunized at 0-, 21-, 28- and 28-day intervals. The antibody was tested by enzyme-linked immunosorbent assay and purified by peptide affinity chromatography (Eurogentec).

##### GCKO-Mice and Characterization of NO-GCβ_1_ Antibody in GCKO Mice

Mice lacking NO-GC (GCKO; genetic background: C57BL/6) were generated as described previously [38]. WT littermates were used as controls. Half the jaw of each mouse (n = 3) was immersion-fixed (in 4% paraformaldehyde + 0.2% picric acid in 0.1 M PBS), decalcified (by 4N formic acid), cryo-protected (in 30% sucrose solution, pH7.4), and cryo-sectioned on a cryostat (at 20 μm). Sections from GCKO and WT mice were incubated with NO-GCβ_1_ antibody at a dilution of 1:2000 using the avidin–biotin–peroxidase complex method (see below).

The animal study protocol was approved by the local animal care committee (Bezirksregierung Unterfranken, Az 55.2 2532-2-1365).

##### Western Blots with NO-GCβ_1_ Antibody Using Human Placenta Tissue Samples

The specificity of the NO-GCβ_1_ antibody was tested in human tissue (placenta) by immunoblot. Briefly, placenta samples (100 μg) were crushed under liquid nitrogen using a mortar. The homogenised samples were suspended in 1000 μL ice-cold lysis buffer (10 mM Tris pH 6.8, 1 mM EDTA, 1% SDS, 0.1% Triton X-100, and 1 mM phenylmethylsulfonyl fluoride, supplemented with complete (Roche, Basel, Switzerland)). After incubation for one hour on ice, the samples were centrifuged at 14,000× *g* for 60 min. The resulting supernatants were immediately frozen and stored at −80 °C until further use. Placenta lysates were loaded onto 10% SDS-acrylamide gels flanked by a protein standard (#26619) (Thermo Fisher Scientific, Darmstadt, Germany), so that the left side was a mirror image of the right side. After electrophoretic separation, half the gel was transferred to a nitrocellulose membrane using a Tobwin transfer buffer system, the other half was stained with Coomassie blue. The former was blocked for 30 min at room temperature (RT) with 5% (*w*/*v*) skimmed-milk powder in PBS containing 0.1% Tween-20. After blocking, the membrane was incubated with rabbit anti-human β_1_-subunit of NO-GC antibody (EP111806: ED11005) at a dilution of 1:3000 overnight at 4 °C. For detection, a horseradish peroxidase-conjugated secondary mouse-anti-rabbit antibody (#211-032-171, Jackson, Lot: 152198) was applied at a dilution of 1:10,000 and incubated for 1 h at RT. Membranes were then processed by chemiluminescence using the ECL Western Blotting Detection System (Amersham Biosciences, Buckinghamshire, UK).

Human placenta tissue samples for immunoblotting were collected at the University Hospital of Cologne. This study was approved by the Ethics Commission of the University of Cologne-Faculty of Medicine (Nr.: 14-244).

##### Mass Spectrometry Analysis

From the Coomassie blue-stained gel, the corresponding band was excised using a scalpel blade and analysed by mass spectrometry (CECAD Proteomics Facility, Cologne, Germany). The analysis was performed on a Q Exactive Plus Orbitrap mass spectrometer that was coupled to an EASY nLC (both Thermo Scientific). Peptides were loaded with solvent A (0.1% formic acid in water) onto an analytical column (2.7 μm Poroshell EC120 C18, Agilent; 50 cm length, 75 μm diameter). Peptides were chromatographically separated at a constant flow rate of 250 nL/min using the following gradient: initial 3% solvent B (0.1% formic acid in 80% acetonitrile), 3–5% B within 1.0 min, 5–30% solvent B within 65.0 min, 30–50% solvent B within 13.0 min, 50–95% solvent B within 1.0 min, followed by washing and column equilibration. The mass spectrometer was operated in a data-dependent acquisition mode. The MS1 survey scan was acquired from 300–1750 m/z at a resolution of 70,000 and 20 ms maximum injection time. The 10 most abundant peptides were isolated within a 1.8 h window and subjected to HCD fragmentation at a normalized collision energy of 27%. The AGC target was set to 5e5 charges, allowing a maximum injection time of 110 ms. Product ions were detected in the Orbitrap at a resolution of 35,000. Precursors were dynamically excluded for 10.0 s.

All mass spectrometric raw data were processed with Maxquant (version 1.5.8.3) [39] using default parameters against the Uniprot canonical human database (UP5640, downloaded 05.08.2020). Follow-up analysis was conducted in Perseus 1.6.15 [40]. Protein groups and identified peptides were filtered for potential contaminants.

### 4.5. Immunohistochemical Methods

#### 4.5.1. Avidin–Biotin–Peroxidase Complex Method

The free-floating frozen sections were treated with 0.3% H_2_O_2_ in 0.05 M Tris-buffered saline (TBS) for 20 min to inhibit endogenous peroxidase. The nonspecific immunoglobulin binding sites were blocked by incubation of sections in blocking solution containing 5% normal goat serum (Vector, Burlingame, CA, USA) and 2% bovine serum albumin (BSA) (Sigma-Aldrich; Taufkirchen, Germany). The sections were incubated overnight with rabbit anti-human polyclonal β_1_-subunits (1:2000) of NO-GC (EP111806: ED11005) at 4 °C. Then, sections were incubated for 1 h with biotinylated goat anti-rabbit IgG (1:1000) (Vector). The sections were incubated for 1 h with avidin–biotin–peroxidase complex (1:100) (Vector) and the immunohistochemical reaction was developed in all incubations for 15 min with 0.05% 3,3′-diaminobenzidine tetrahydrochloride (Sigma-Aldrich) in 0.05 M Tris-HCl buffer, pH 7.6, containing 0.01% H_2_O_2_ and 0.01% nickel sulfate.

In order to test the specificity of the secondary antibodies and the avidin–biotin–peroxidase reagents (NGS, BSA, avidin–biotin–peroxidase complex), the primary antibodies were omitted from the immunohistochemical incubations.

#### 4.5.2. Immunofluorescence

Free-floating sections were incubated with 5% normal goat serum + 2% BSA to block nonspecific immunoglobulin binding sites. In separate incubations, the sections were incubated with rabbit polyclonal anti-human α_1_-subunits (1:1000) and rabbit polyclonal β_1_-subunits (1:1000) of NO-GC antibodies overnight at 4 °C. The sections were incubated for 1 h with the DyLightTM 550-conjugated goat anti-rabbit IgG (Thermo Fischer Scientific). To show the cell nuclei, sections were stained with DRAQ5 (Cell Signaling Technology, Frankfurt am Main, Germany) for 15 min. The sections were coverslipped with Aqua Poly/Mount (Polysciences Inc., Warrington, PA, USA) and analyzed with an LSM510 confocal microscope (Carl Zeiss, Jena, Germany).

To exclude the presence of nonspecific labeling of secondary antibodies and immunohistochemical reagents (NGS, BSA), negative controls were performed by omitting the primary antibody.

#### 4.5.3. Double Immunofluorescence

Free-floating sections were incubated with 5% normal goat serum + 2% BSA to block nonspecific immunoglobulin binding sites. Firstly, these sections were incubated for 1h with mouse anti-NF200 (1:4000, Sigma Aldrich) overnight at 4 °C, and after, with DyLightTM 488-conjugated goat anti-mouse IgG (Thermo Fischer Scientific). Then, they were incubated with rabbit polyclonal anti-human β_1_-subunits (1:2000) overnight at 4 °C, and after, for 1h with the DyLightTM 550-conjugated goat anti-rabbit IgG (Thermo Fischer Scientific). To visualize nuclei, these sections were stained for 15 min with DRAQ5 (Cell Signaling Technology). At last, they were mounted with Aqua Poly/Mount (Polysciences Inc., Warrington, PA, USA) and analyzed with an LSM510 confocal microscope (Carl Zeiss).

To exclude the presence of nonspecific labeling of secondary antibodies and immunohistochemical reagents (NGS, BSA), negative controls were performed by omitting the first and second primary antibodies.

### 4.6. Fluorescence Intensities of the NO-GCα_1_ and NO-GCβ_1_ in Pericytes and Statistical Analysis

The fluorescence intensities of the NO-GCα_1_ and NO-GCβ_1_ antibodies in pericytes of the decalcified (*n* = 6) and non-decalcified (*n* = 3) molars were measured in QuPath. For this, the whole cells were manually annotated, and the mean was used to measure the fluorescence intensity with a scan of 1 μm^2^. The results were exported for further statistical analysis.

Statistical analysis was performed with R (version 4.3.0). Measurements were compared using a two-tailed unpaired *t*-test. The normal distribution was tested using the Shapiro–Wilk test. Plots were generated with ggplot.

## Data Availability

The data presented in this study are available on request from the corresponding author.

## Supplementary Materials

The following supporting information can be downloaded at https://www.mdpi.com/article/10.3390/ijms26010030/s1.
