# Supplementary material for: The α1- and β1-Subunits of Nitric Oxide-Sensitive Guanylyl Cyclase in Pericytes of Healthy Human Dental Pulp
Source: Int J Mol Sci. 2024 Dec 24;26(1):30. doi: 10.3390/ijms26010030 (PMC11720548; doi:10.3390/ijms26010030)
Supplement: Supplementary file 1 [file ijms-26-00030-s001.zip › ijms-3352341-supplementary.pdf]

Supplementary Materials

Results

1. Characterization of healthy human dentin–pulp complex

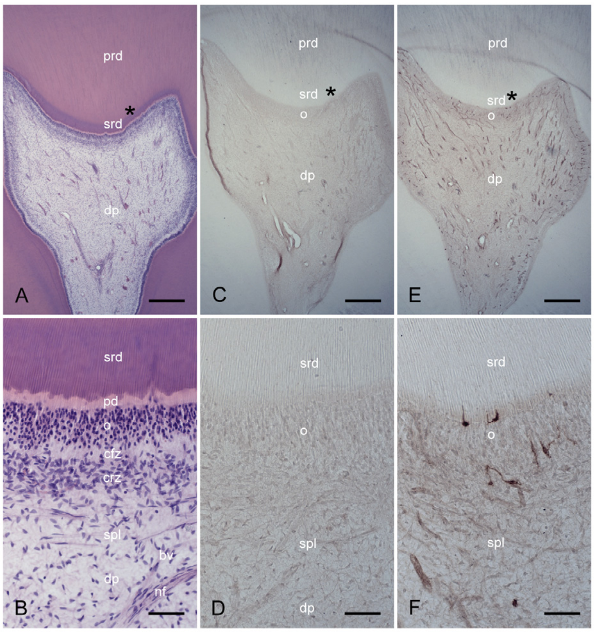

Figure S1. Histological characterization of the healthy dentin-pulp complex by HE-staining and expression of MCT and HLA-DR in cells from consecutive sections of a human molar. The overview image of the healthy molar showed the structural order of the dentin-pulp complex with primary dentin (prd) (A). In detailed image from the region marked with an asterisk in A, secondary dentin (srd), predentin (pd), odontoblast layer (o), cell-free (cfz) and cell-rich (crz) layers as well as subodontoblastic plexus (spl) with nerve fibres and blood vessels were observed in a cellular order (B). In the overview (C) and in the detail (the region marked in C with asterisk; D) images of following section, no immunoreactivity for MCT was detected in cells of the healthy dental pulp. Images of the next consecutive section showed clear HLA-DR signals in several cells of healthy dental pulp in the overview (E) and detail images (the region marked in E with asterisk; F). Scale bars: (A, C, E) = 500 µm; (B, D, F) = 60 µm.

2. Mass spectrometry analysis of NO-GCβ1 Antibody

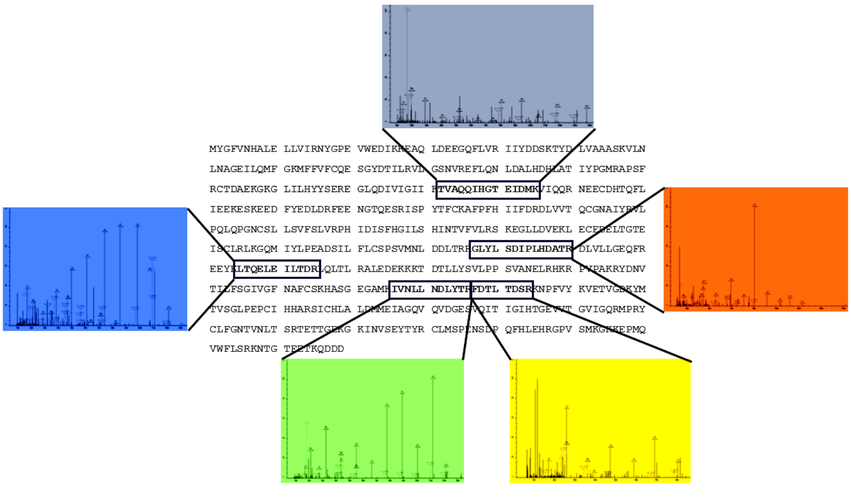

Figure S2. Mass spectrometry analysis of excised immunoblot bands obtained from human placenta tissue extracts using polyclonal rabbit anti-human NO-GCβ1 antibody. The antibody against the

human β_1-subunit of NO-GC was generated in a rabbit by immunization with a synthetic peptide (EP111806: ED11005) containing the C-terminal domain (CSRKNTGTEETKQDDD) of the human β_1-subunit (Eurogentec). The excised band of immunoblot revealed 5 peptides of the β_1-subunit of NO-GC, corresponding to a coverage of 9.4% of the β_1-subunit of the NO-GC protein.

3. Co-localization of NO-GCβ_1 with NF200 in nerve fibers of the decalcified human dental pulp

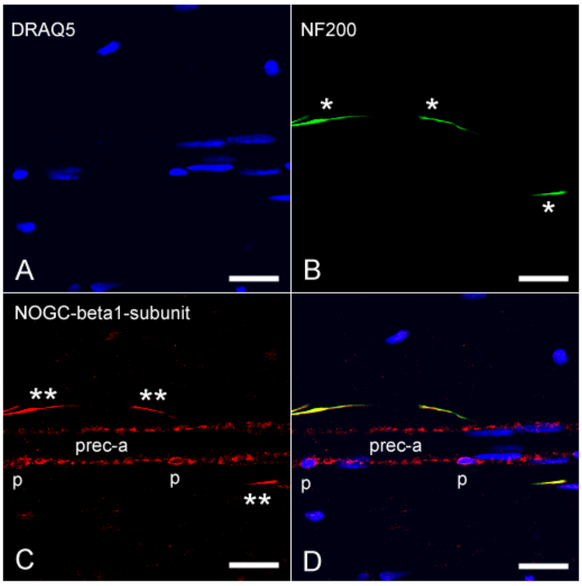

Figure S3. Co-localization of NO-GCβ_1 with NF200 (a marker for myelinated Aβ and Aδ nerve fibers) in nerve fibers of healthy human dental pulp. The cell nuclei were vizualized by DRAQ5 staining (A). Nerve fibers of the dental pulp were detected by NF200 (green; single asterisks) (B). NO-GCβ_1 (red) is expressed in precapillary arterioles (pre-a), in pericytes (p) and in nerve fibers (two asterisks) (C). The overlay shows co-localization of NO-GCβ_1 with NF200 (yellow) in nerve fibers of human dental pulp (D). Scale bars: (A-D)= 20 µm.

4. Immunohistochemical controls of the avidin-biotin-peroxidase complex method

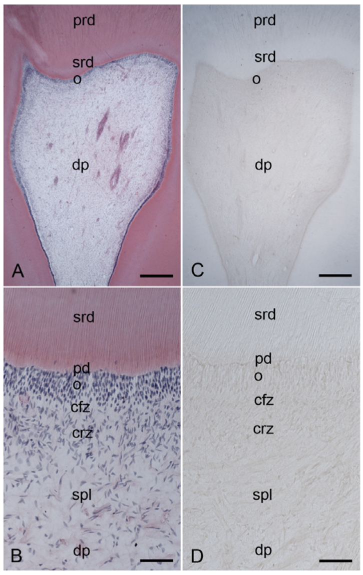

Figure S4. Immunohistochemical control incubation for the avidin-biotin-peroxidase complex method without primary antibodies. Healthy human dental pulp was characterized with HE staining (A, B). Incubation without primary anti-human NO-GCβ_1 antibodies (EP111806: ED11005) resulted in no specific immunostaining (C, D). Instead, only normal background staining due to the

DAB substrate was visible (C, D). prd: primary dentin, srd: secondary dentin, pd: predentin, o: the odontoblast layer, cfz: cell-free zone, crz: cell-rich zone, spl: the subodontoblastic plexus, dp: dental pulp. Scale bars: (A, C)= 500 μm, (B, D)= 60 μm.
